# Mumio (Shilajit) as a potential chemotherapeutic for the urinary bladder cancer treatment

**DOI:** 10.1038/s41598-021-01996-8

**Published:** 2021-11-19

**Authors:** T. Kloskowski, K. Szeliski, K. Krzeszowiak, Z. Fekner, Ł. Kazimierski, A. Jundziłł, T. Drewa, M. Pokrywczyńska

**Affiliations:** 1 Department of Regenerative Medicine, Cell and Tissue Bank, Chair of Urology and Andrology, Collegium Medicum, Nicolaus Copernicus University, M. Sklodowskiej-Curie 9, 85-094 Bydgoszcz, Poland; 2 Department of Tissue Engineering, Chair of Urology and Andrology, Collegium Medicum, Nicolaus Copernicus University, Bydgoszcz, Poland; 3 Department of Plastic, Reconstructive and Aesthetic Surgery, Collegium Medicum, Nicolaus Copernicus University, Bydgoszcz, Poland; 4 Chair of Urology and Andrology, Collegium Medicum, Nicolaus Copernicus University, Bydgoszcz, Poland

**Keywords:** Bladder cancer, Natural products, Chemotherapy

## Abstract

Mumio (Shilajit) is a traditional medicinal drug known and used for hundreds of years. Bladder cancer is one of the most common cancer types and better treatments are needed. This study analysed the in vitro effect of Mumio on urinary bladder cancer cells (T24 and 5637) in comparison to normal uroepithelial cells (SV-HUC1). Cytotoxicity of Mumio was analysed in these cell lines via MTT and real-time cell growth assays as well via the assessment of the cytoskeleton, apoptosis, and cell cycle. Mumio affected the viability of both cell types in a time and concentration dependent manner. We observed a selectivity of Mumio against cancer cells. Cell cycle and apoptosis analysis showed that Mumio inhibited G0/G1 or S phase cell cycle, which in turn induced apoptosis. Our results showed that Mumio was significantly more cytotoxic to urinary bladder cancer cells than to normal cells. These results are promising and indicate Mumio as a great candidate for urinary bladder cancer treatment and further investigations should be performed.

## Introduction

Mumio has been known and used in ancient medicine for more than 4000 years and occupies the most prestigious place among traditional medicinal drugs. Mumio is a pale-brown to a black-brown tar-like substance formed over a hundred years from mineral rock and plant remains^1, 2^. Different names are used depending on where it is found such as: Mumijo, Mumie (Russia), Saljit, Shilajit (India), Kao-Tun (Birma), Arakul Dshabal (Kyrgyzstan) and many others^3^. Raw Mumio can be found in places over 1000 m above the sea level such as caves, crevices and rock cavities, in the form of infiltrations or clusters of icicles in dry places that are sheltered from wind and sun^4^. It has been found as crusts in rock cracks or interstices frequently in the Himalayas and mountainous areas in southern Kazakhstan. It is also found in the alpine region of Central Asia: Afghanistan, Nepal, Bhutan, Pakistan, China, Tibet, and the former U.S.S.R^4, 5^. In India Mumio is used as a remedy for skin diseases, injuries, dislocations and bone fractures and for the treatment of osteoporosis. Mumio is considered to improve memory and to inhibit aging of the brain through its neuroprotective activity. Its mechanism of action is based on fulvic acid-mediated prevention of Tau self-aggregation. Because of these effects it became relevant to diseases of the peripheral nervous system such as neuralgia, radiculitis, prevention of Alzheimer`s disease or amnesia^6–8^. Due to its high iron content and antioxidant properties, Mumio is also used to treat anaemia^4, 9^. Mumio is also recommended for patients with diabetes, digestive disorders, tuberculosis, chronic bronchitis and asthma^6, 7, 10, 11^.

Mumio from different regions of the world have similar physical properties and similar qualitative chemical compositions. However, the proportions of the individual constituents between the samples from different regions differ^12^. Based on its origin, Mumio is divided into three types: the Mumie of petroleum, which is formed through transformations of deep oil products from the mountains, plant Mumie (mummies-Asil) and Mumie-Kiem, which are formed from long-term humification of alpine rodents^2, 4^.

The origin of Mumio is a hot topic of discussion among scientists. There are two theories of its origin: plant and animal^10^. Mumio contains trace amounts of soil and organic alkaloids, which suggest a plant origin. On the other side, the presence of hypuronic acid and albumin suggests an animal origin. It has been shown that Shilajit consists mainly of organic matter (60–80%) that is derived from fossil vegetation, which has been compressed under the layers of rocks under high temperature and pressure over several centuries of metamorphosis. The remaining 20–40% of Mumio are different kinds of mineral matter, and ~ 5% are trace elements^2, 4, 12^.

Extensive studies were conducted to determine the exact chemical nature of Mumio by Kong et al. in 1987 and Ghosal et al*.* in 1988. These studies found that the main components of Mumio are humus (60–80%), benzoic acid, fatty acids, ichthyol, ellagic acid, resin, triterpenes, sterol, aromatic carboxylic acids, bioactive 3,4-benzokoumarins, amino acids, phenolic lipids and microelements. The most important role among these have the following bioactive molecules: dibenzo-alpha-pyrones, humic acid and fulvic acid. These components mediate Mumio’s strong antioxidant activity and are carriers for active ingredients. The studies carried out by Ghosal et al. showed that these acids inhibit lipid peroxidation and possess the ability to recycle ascorbic acid and thereby exhibiting significant antioxidant activity^7, 11, 13–15^. Mumio is also a rich source of amino acids, especially exogenous, such as methionine, leucine and threonine, and also endogenous such as histidine, proline, glycine, tyrosine, arginine and aspartic acid^9, 10^.

Cancer is one of the most common diseases in this day and age. Despite the development of new and improved treatment and diagnosis methods, it is still the second most common cause of deaths. In 2018, urinary bladder cancer was the sixth most common cancer among men, and seventeenth among women^16^. About 90% of patients are over 55 years old. The treatment methods depend on the cancer stage in bladder cancer. The most commonly used methods are surgical: transurethral resection of bladder tumour (TURBT) for non-invasive tumours and radical cystectomy for invasive tumours. Both of these surgical procedures are usually combined with either chemotherapy, radiotherapy or immunotherapy, to increase the probability for complete depletion of the carcinoma cells^17^.

**Figure 1:**
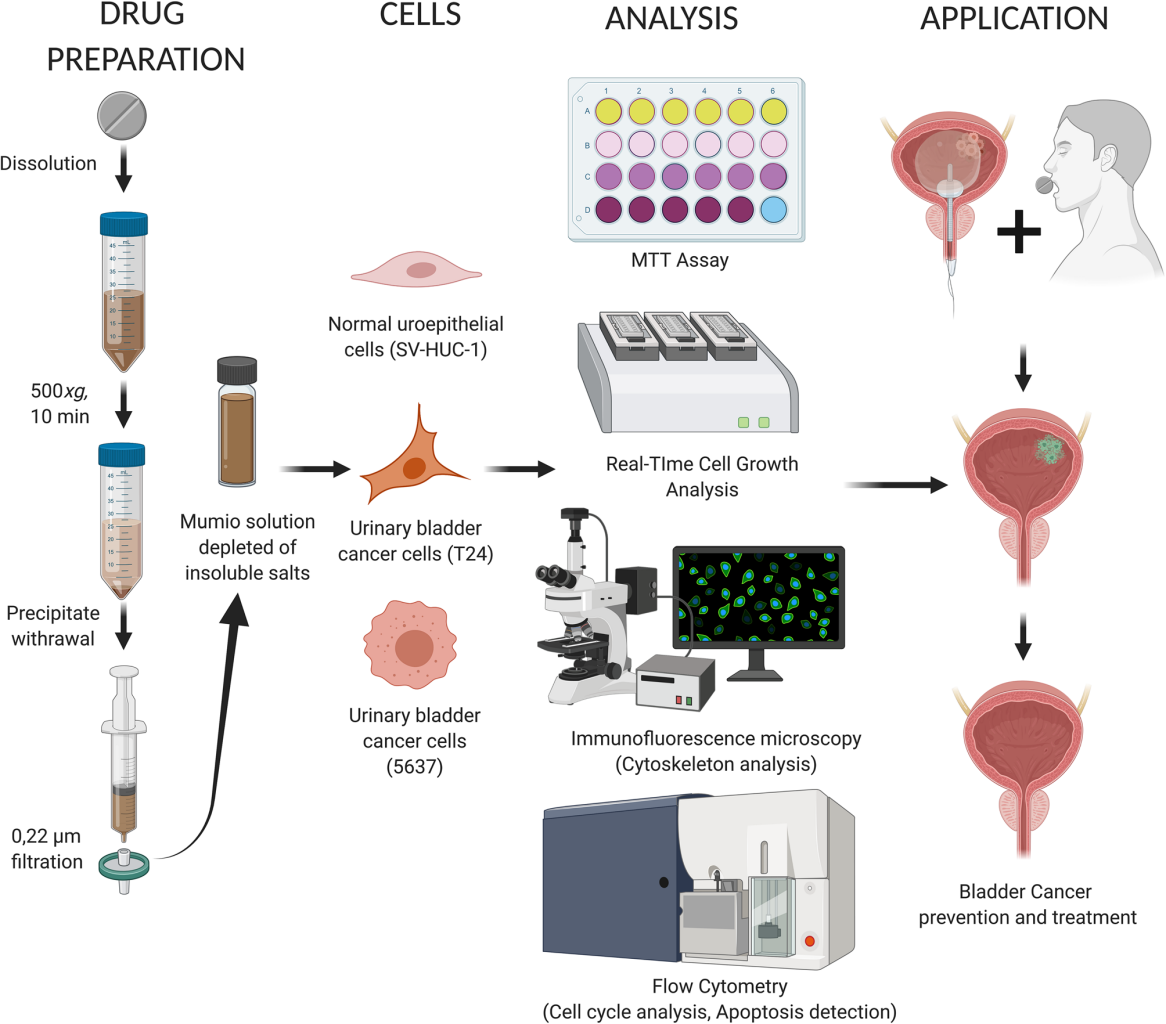
Workflow of the methods used to examine how Mumio affects normal and urinary bladder cancer cells. Created with BioRender.com.

Based upon the aforementioned properties of Mumio, we hypothesized that administration of this substance may improve the effectiveness of bladder cancer therapy or that it can be used as an adjuvant to support current therapies. Thus this study investigated the effects of Mumio on urinary bladder cancer cells (T24 and 5637) in comparison to normal uroepithelial cells (SV-HUC-1, Fig. 1).

## Results

### Cell morphology

**Figure 2:**
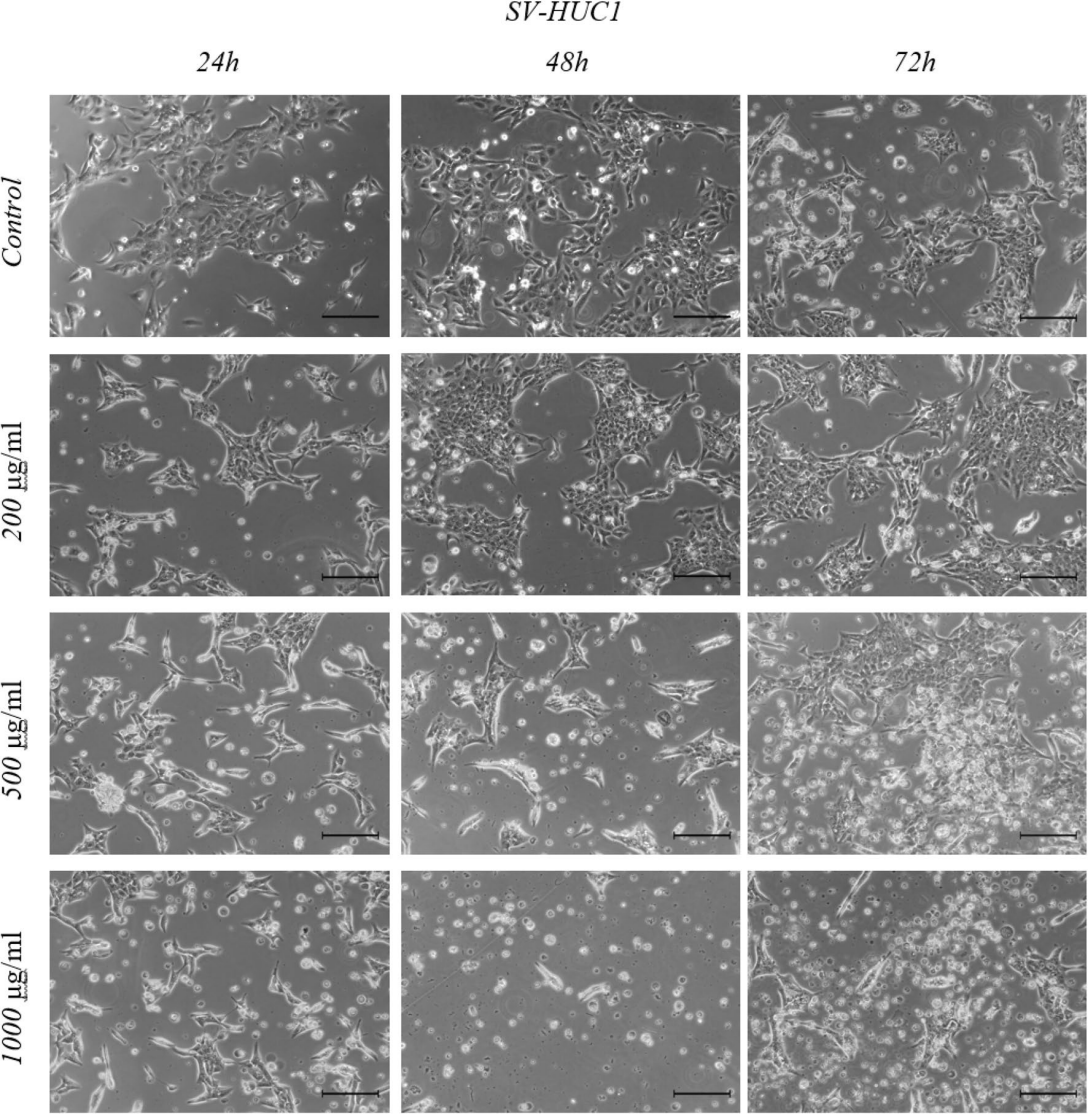
SV-HUC-1 cell morphology after Mumio treatment. Fewer attached cells were observed after 24 h incubation at the highest Mumio concentration. Changes in cell morphology such as cell shrinkage and increased cell detachment were observed after 48 and 72 h. Light microscope (20×, bar 100 μm) SV-HUC-1—Normal uroepithelial cell line.

**Figure 3:**
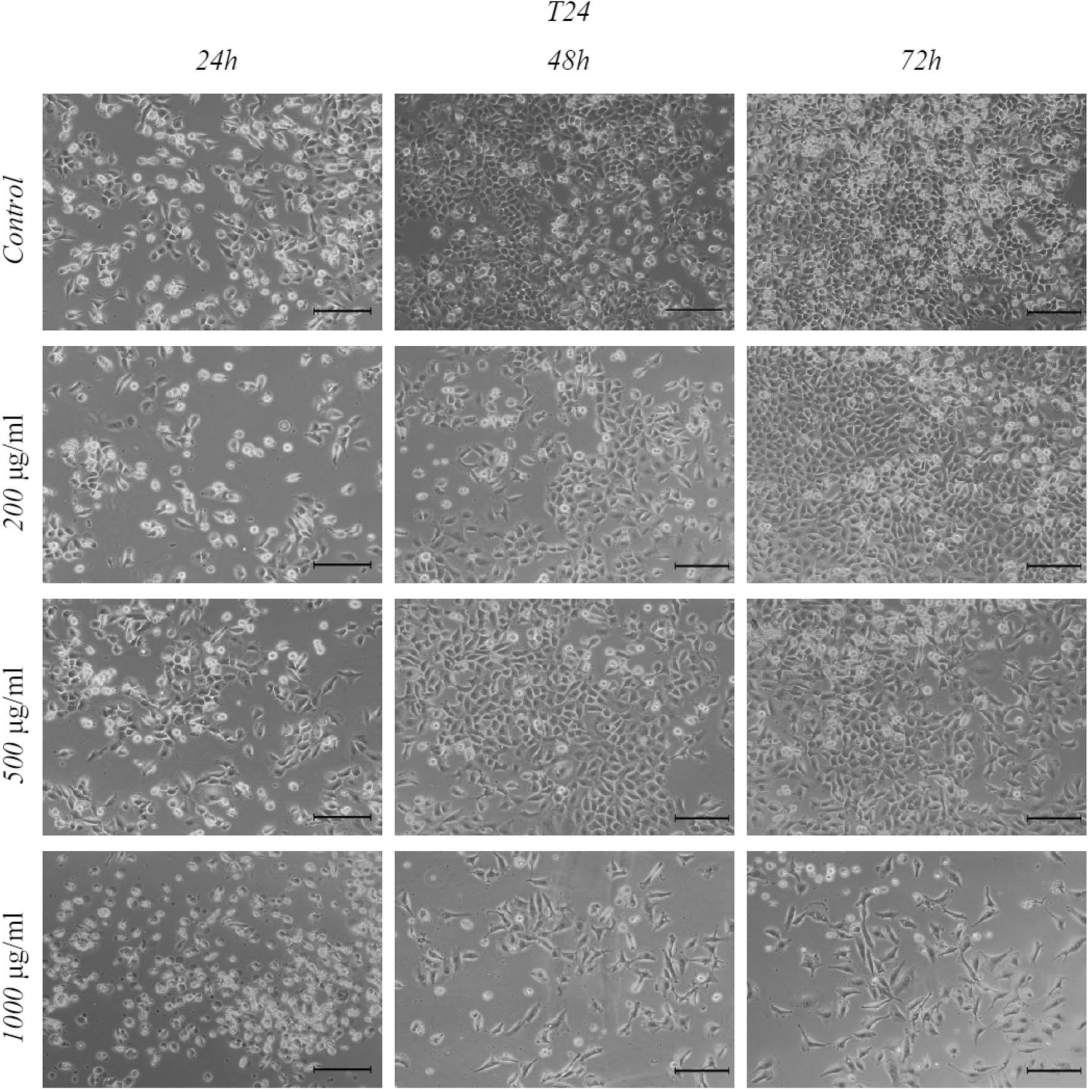
T24 cell morphology after Mumio treatment. Mumio at higher concentrations caused cell shrinkage, lower cell number, and increased cell detachment. Light microscope (20×, bar 100 μm), T24—Transitional Urinary Bladder Carcinoma Cell Line.

**Figure 4:**
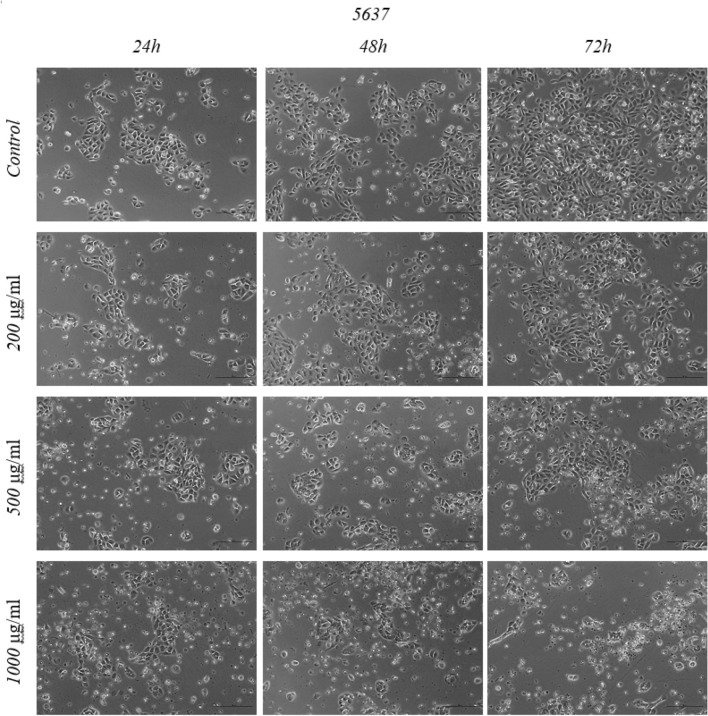
5637 cell morphology after Mumio treatment. Increased cell detachment was noted at 500 μg/ml and higher concentrations after 24 h. Cell morphology was changed as indicated by cell shrinkage and increased cell detachment. These effects were observed after 48 and 72 h. Light microscope (20×, bar 100 μm). 5637—grade II urinary bladder carcinoma cell line.

We did not observe any changes in cell morphology after 24 h incubation in SV-HUC-1 cells. We only observed a lower number of attached cells at the highest concentrations of Mumio. After 48 h incubation 200 μg/ml of Mumio affected the cells, an effect that increased with increasing drug concentrations. Cell shrinkage, a lower cell number and rounding were observed at 500 μg/ml. After 72 h of drug exposure, cells were affected at 200 μg/ml (Fig. 2). Cell number and morphology was affected at 200 μg/ml in T24 cells at all studied time points. Cell shrinkage, rounding and a lower cell number were observed with increasing concentrations of Mumio (Fig. 3). An increased number of round, detached cells was observed with 500 μg/ml and higher concentrations in 5637 cells. A similar trend was noted after 48 h with a striking difference at 500 and 1000 μg/ml and after 72 h fewer cells were present at 200 μg/ml. At 1000 μg/ml after 72 h incubation most of the 5637 cells lost their normal morphology, and a considerable amount of cell debris was observed (Fig. 4).

### Cell viability and selectivity index

**Figure 5:**
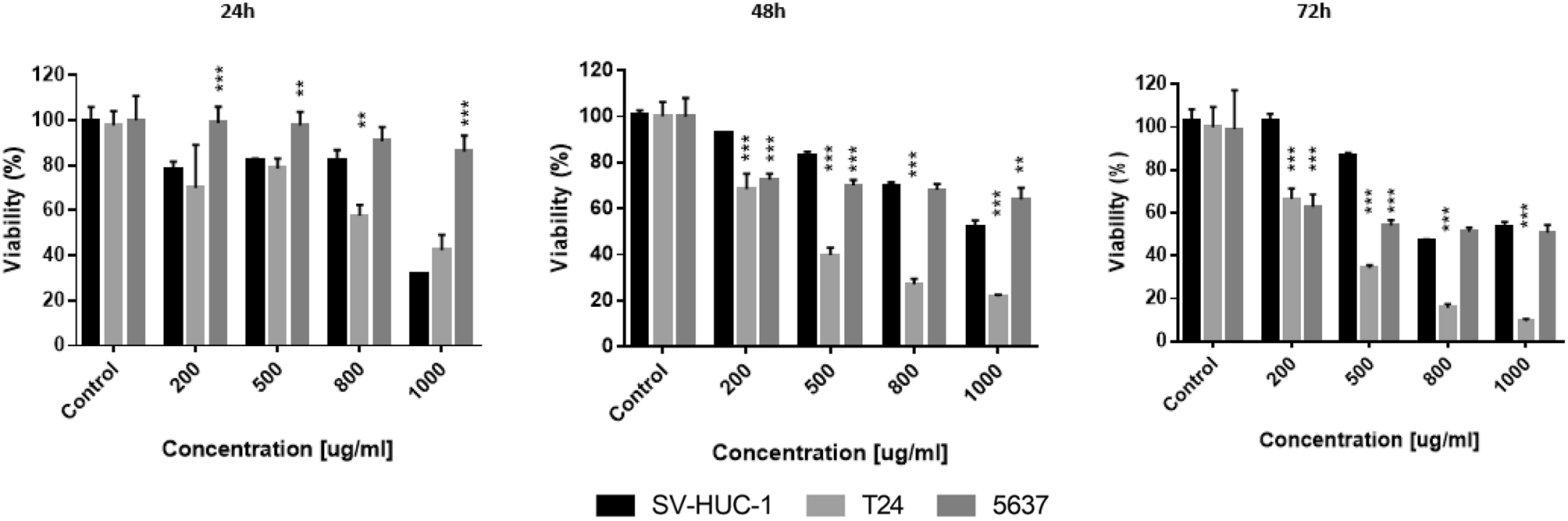
Cytotoxic properties of Mumio on SV-HUC1, 5637 and T24 cells. Results were obtained via MTT assay. Viability of SV-HUC-1, T24 and 5637 cells was affected in a time and concentration dependent manner by Mumio. *p < 0.05; **p < 0.01; ***p < 0.001 (T24 and 5637 compared to SV-HUC-1). SV-HUC-1—normal urothelial cell line, T24—transitional urinary bladder carcinoma cell line, 5637—grade II urinary bladder carcinoma cell line.

Mumio was cytotoxic in T24 cells in a time and concentration dependent manner. There were no significant differences in cells viability after 24 h at 200, 500 and 800 μg/ml Mumio in SV-HUC-1 cells. However, cell viability at all tested concentrations was lower compared to control. A significant difference was also noticed at 1000 μg/ml. Comparison between SV-HUC-1 and cancer T24 cell line after Mumio treatment showed that normal cells at all tested concentrations after 48 and 72 h were significantly lesser effected. After 24 h of treatment a significant difference between SV-HUC-1 and T24 cells was only noticed at 800 μg/ml Mumio. 5637 cancer cells were significantly more viable than SV-HUC-1 at all tested Mumio concentrations after 24 h. The cancer cells were significantly lesser affected at 200 and 500 μg/ml Mumio after 48 h of incubation, while the opposite was observed at 1000 μg/ml. A similar ratio as seen after 48 h at 200 and 500 μg/ml was observed after 72 h (Fig. 5).

MTT assay enabled the calculation of the lethal concentration (LC). The obtained values were used in further analysis (Table 1). All calculated LCs of Mumio were higher for the normal cell line (SV-HUC-1) than for cancer cells (T24). However, 5637 cells had higher LC values than normal cells after 24 and 48 h exposures. Only at the 72 h time point were the LC concentrations higher for cancer cells than for normal cells. Calculated with the obtained LC_50_ values, selectivity indexes showed that Mumio is selectively toxic against T24 cell after 48 and 72 h of incubation, with a 24 h SI value very close to 2.0. SI values were lower than 1.0 for the 24 and 48 h time points, and 1.2 for the 72 h time point in the 5637 cells. For both cell lines SI values increased with prolonged incubation time.

**Table 1:** Lethal concentrations of Mumio in SV-HUC-1, 5637 and T24 cells, after 24, 48 and 72 h exposures and selectivity indexes for T24 and 5637 cells.

Concentration (µg/ml)	SV-HUC-1	T24	T24 Selectivity Index	5637	5637 Selectivity Index
**24 h**					
LC_10_	278	142	1.96	840	0.49
LC_50_	1829	934		3740	
LC_90_	6075	3103		6640	
**48 h**					
LC_10_	181	66	2.74	211	0.85
LC_50_	1193	436		1390	
LC_90_	3963	1448		4610	
**72 h**					
LC_10_	159	45	3.47	132	1.2
LC_50_	1045	301		866	
LC_90_	3473	999		2880	

All LCs were lower for T24 cells, indicating a higher sensitivity for Mumio. SV-HUC-1—normal urothelial cell line, T24—transitional urinary bladder carcinoma cell line, 5637—grade II urinary bladder carcinoma cell line, LC—lethal concentration.

### Real-time cell growth analysis

**Figure 6:**
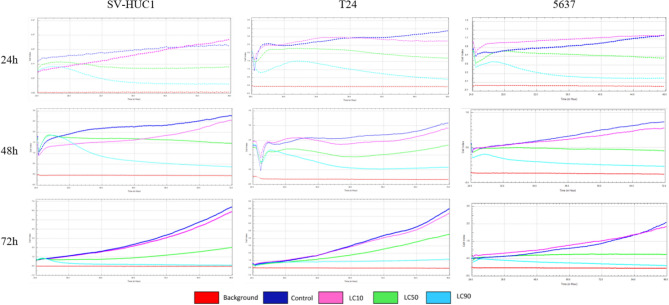
Real-time cell analysis after exposure to the calculated lethal concentrations of Mumio for 24, 48 and 72 h. Our results confirmed the LCs calculated with MTT for all cell lines for all three incubation times. The charts represent readings in 15 min intervals after the addition of Mumio. SV-HUC-1—normal urothelial cell line, T24—transitional urinary bladder carcinoma cell line, 5637—grade II urinary bladder carcinoma cell line, LC—lethal concentration.

The results obtained with xCELLigence RTCA DP system confirmed that the lethal concentrations acquired via the MTT assay caused the decrease of viable cells by 10, 50 and 90%. Results were confirmed for both cell lines for all three incubation times (Fig. 6).

### F-actin staining

**Figure 7:**
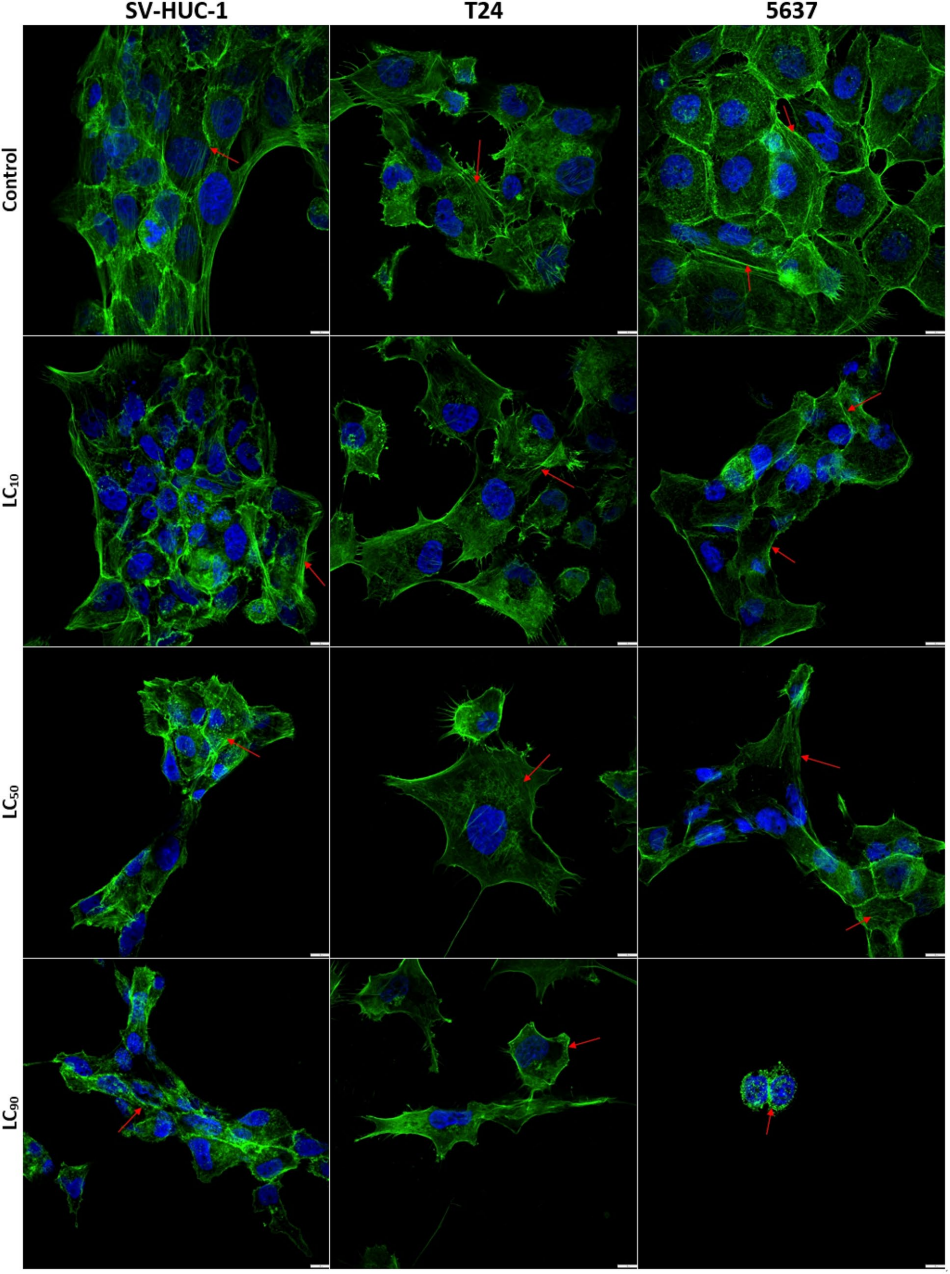
SV-HUC1, T24 and 5637 cells stained with Alexa Fluor 488 phalloidin (green) and DAPI (blue) after exposure to the corresponding 24 h lethal concentrations of Mumio. Degradation of stress fibres was noticed after exposure to Mumio in all tested cell lines. Fluorescent microscopy (100×, bar 10 μm). SV-HUC-1—normal urothelial cell line, T24—transitional urinary bladder carcinoma cell line, 5637—grade II urinary bladder carcinoma cell line, LC—lethal concentration.

Staining of F-actin microfilaments revealed that Mumio at LC_10_ in SV-HUC-1 cells did not cause any significant reorganization of the actin filaments. However, the LC_50_ concentration caused degradation of stress actin fibres indicated by increased round aggregates of actin, while at LC_90_ almost no stress fibres were observed. A similar trend was observed in T24 cells, with no effect at LC_10_, noticeable smaller stress fibre at LC_50_, and round actin aggregates instead of fibres at LC_90_. However, the cell shape was preserved. Incubation at LC_10_ caused a noticeable lower number of long stress actin fibres in 5637 cells. After exposure to LC_50_, the stress fibres had a less organized structure and more short fibres were present. Exposure with Mumio at LC_90_ completely changed the cell morphology and only round actin aggregates were observed (Fig. 7).

### Cell cycle analysis

**Figure 8:**
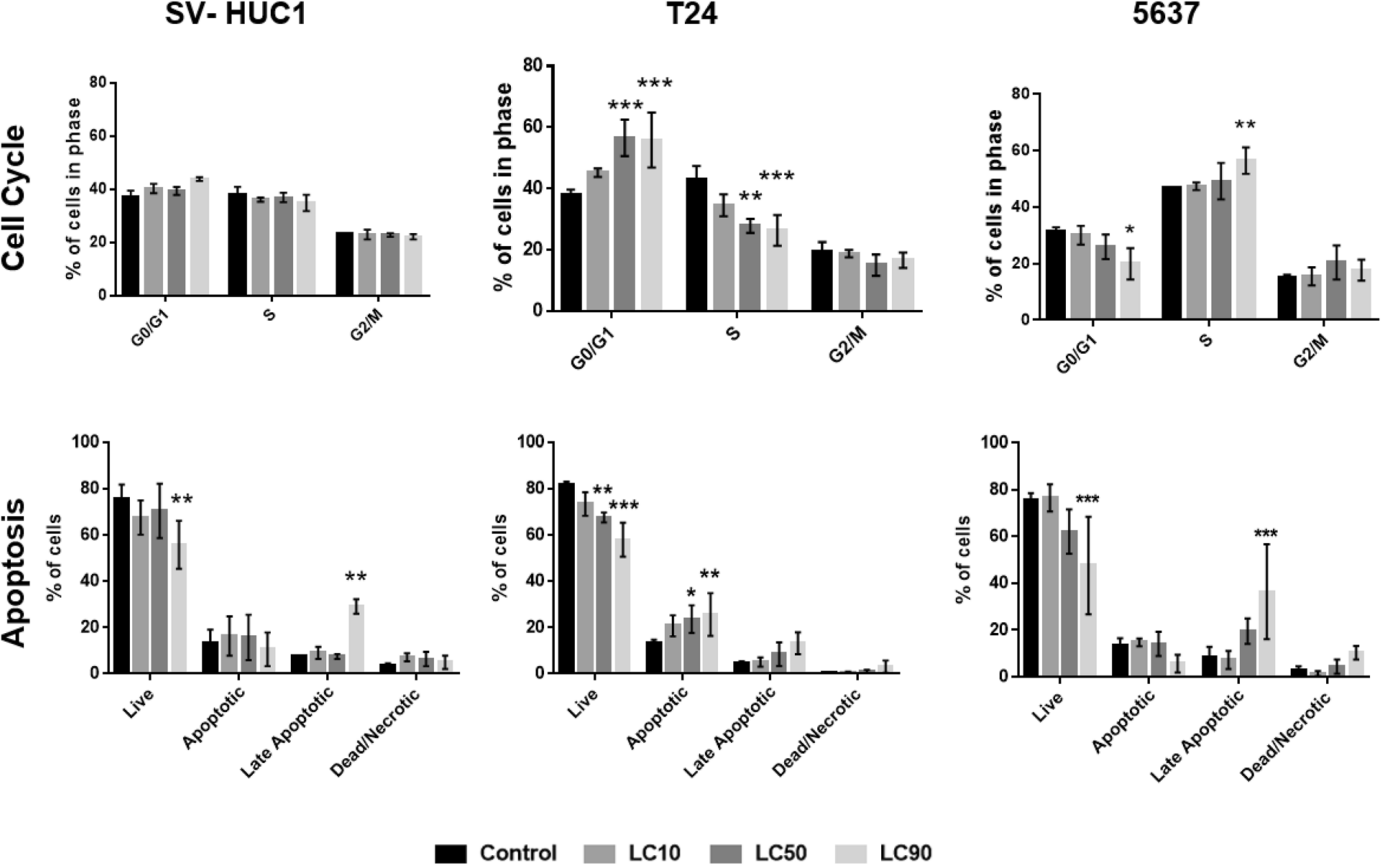
Cell cycle and apoptosis by flow cytometry after cell exposure to their 24 h lethal concentrations of Mumio. Mumio did not affect the cell cycle of SV-HUC1 cells. Increased G0/G1 and decreased S phase cell populations were observed after exposure of T24 cells to LC_50_ and LC_90_. Increased cell numbers in G0/G1 phase and S phase were noted in 5637 cells. Apoptosis analysis revealed significantly fewer viable cells with increased late apoptotic cells after exposure to LC_90_ of Mumio in SV-HUC1. Significantly fewer viable cells with increased apoptotic cells were observed in T24 cells after incubation with LC_50_ and LC_90_ of Mumio. SV-HUC-1—normal urothelial cell line, T24—transitional urinary bladder carcinoma cell line, 5637—grade II urinary bladder carcinoma cell line, LC-lethal concentration,*p < 0.05; **p < 0.01; ***p < 0.001 (compared to control).

Cell cycle analysis revealed no significant changes in cell cycle distribution in SV-HUC1 cells after exposure LCs of Mumio for 24 h. A significant increase in G0/G1 cells in the LC_50_ and LC_90_ treatment groups was observed in T24 cells (*p* < 0.001). Additionally, a significant decrease in cells in the S phase was observed in LC_50_ (*p* < 0.01) and LC_90_ (*p* < 0,001) with no significant change in the G2/M cell number. Significantly fewer cells were in G0/G1 phase and significantly more cells were found in S phase in 5637 cells after exposure to LC_90_ concentration of Mumio and no significant changes were observed at lower concentrations (Fig. 8).

### Apoptosis

Analysis of apoptosis revealed increased late apoptotic cells in SV-HUC-1 treatment group after exposure to LC_90_ with a significant decrease in viable cells (*p* < 0.01) when compared to control. No effect was observed at lower concentrations. A significant increase in apoptotic cells was noticed after Mumio exposure at LC_50_ (*p* < 0.05) and LC_90_ (*p* < 0.01) in T24 cells. This occurred along with a significant decrease viable cells. 5637 cells treated with LC_90_ had significantly fewer viable cells (*p* < 0.001) along with a significant increase in late apoptotic cells (*p* < 0.001). There were no significant changes at the lower concentrations (Fig. 8).

## Discussion

Numerous studies have confirmed the widespread use of Mumio in traditional medicine mainly because of its anti-ulcerogenic, anti-inflammatory, antioxidant, immunomodulatory, memory enhancement and anxiolytic activities^8, 11^. A clinical randomized double-blind placebo-controlled trial conducted by Sadeghi et al*.* found a beneficial effect of oral consumption of 500 mg Mumio capsules in the healing of Tibia fracture. The time to full recovery was significantly shorter and no increased adverse effects^18^. This study indicates that the oral consumption of 500 mg of Mumio does not cause adverse effects, and can be considered for application to other diseases.

Mumio is an adaptogen used for urinary tract problems, sexual dysfunctions and improved prostate health. A study in 1997 showed that Mumio has a beneficial effect on bladder obstruction in patients with benign prostate hypertrophy^6, 11, 19^. Mumio has antimicrobial activity against various strains of pyogenic microorganisms, which is associated with the presence of benzoic and fulvic acid. These antibacterial properties of Mumio can be supportive for bladder cancer treatment and prevention according to the two-hit hypothesis as it not only impairs cancer cell growth but also prevents chronic inflammation, which are considered to be important factors in initiation and progression of bladder cancer^20^. Ellagic and tannic acids are natural polyphenolic antioxidants that inhibit superoxide anion radical production and phospholipase A2 activity. Khanduja et al. and Thresiamma et al*.* showed that these acids have anticarcinogenic and radioprotective properties^21, 22^. In 1991, Ghosal et al*.* found that Mumio extract significantly inhibits cancer cell proliferation in Ehrlich ascites^4^. Moreover, safety of long-term administration of Mumio was evaluated in a rat model by Velmurugan et al*.* They showed that daily Mumio administration for 91 days caused only minor changes in the liver of those animals that received the highest concentration of Mumio. These changes correlated with an excess amount of iron in the liver showing potential mechanism of liver changes development^9^.

To date only a few studies evaluated effect and the mechanisms of action of Mumio on cancer cells. Pant et al. showed that Mumio inhibits growth of hepatocellular Huh-7 cancer cell line and induces apoptosis in vitro^23^. In our study a significant increase in apoptotic cells was also observed after incubation of T24 cancer cells with Mumio at its LC_50_ and LC_90_. Additionally, late apoptotic cells were also increased after exposure to Mumio at its LC_90_ in 5637 cancer cells. In SV-HUC1, normal uroepithelial cells, increased late apoptotic cells were only observed after incubation with Mumio at its LC_90_. Moreover, a significant increase of cells in G0/G1 phase with decreased cells in S phase was observed after exposure to Mumio at LC_50_ and LC_90_ concentrations, indicating G0/G1 cell cycle arrest in T24 cells. In 5637 cells, the cell cycle was differently dysregulated such that fewer cells were found in G0/G1 phase and more cells were found in S phase. The cell cycle distribution after exposure to Mumio was unaffected in SV-HUC1 cells. These differences indicate that Mumio has different mechanisms of action in these two cancer cell lines. The different mechanisms of action and activity may be due to different proliferation and metabolism rates of these two cancer cell lines.

Thawatchai et al*.* analysed the effect of Mumio on six different human cancer cell lines: breast (MDA-MB-231), lung (A549), liver (HepG2), colorectal (SW-620), ovary (SKOV-3), and cervix (Hela) in comparison to the normal human lung fibroblast cell line MRC-5. They found that Mumio exhibited a stronger effect on almost all tested carcinoma cell lines, except the breast cancer cell line, when compared to normal lung fibroblast line^24^. We also evaluated different cell types, and found similar effects. We found stronger cytotoxicity in one of the tested cancer cell lines when compared to normal cells of the same organ. A direct comparison to our results is not possible because we used lower concentrations of Mumio (25–200 μg/ml). Jafari et al*.* assessed the effect of Mumio on breast cancer cells, MCF-7, and lung carcinoma cell lines, A549. They found a concentration dependent cytotoxic effect of Mumio on both cancer cell lines. Their results in the A549 cells were similar to those found by Thawatchai et al*.*^25^. In contrast to these studies, our study assessed the effect of Mumio between normal and cancer cells of the same organ. This approach enables a better assessment of the drug effectivity and safety at the initial research stage. All LC values obtained in this study were higher for normal cells than for T24 cancer cells. Moreover, the calculated selectivity index for T24 after 48 and 72 h exposures were higher than 2.0 indicating a high selectivity for cancer cells. However, in 5637 cancer cells only Mumio exposures for 72 h were lower than normal cells, and the SI was not higher than 2.0 indicating general toxicity. On the other hand the SI increased in both cell lines with prolonged incubation, indicating a potential benefit of long term administration of Mumio in the treatment and prevention of bladder cancer.

Mumio also affected the cytoskeleton of these two selected cancer cell lines. The actin filaments were damaged but spread evenly while the cell shape was preserved at the LC_90_ concentration in T24 cells. However, the 5637 cells rounded up and the actin filaments were concentrated as round aggregates. Additionally, Mumio affected the cell cycle differently in these two cancer cell lines such that the cell cycle was arrested in different phases. T24 cells were arrested in G0/G1 cell cycle while in 5637 cells were inhibited in S phase. This phenomenon may be explained by the multicomponent composition of Mumio as different components can affect cells with a different metabolism and proliferation rates. This suggests that potential long-term administration of Mumio may be beneficial in the prevention of development or relapse of urinary bladder cancer. Our research showed that Mumio at the tested concentrations has a stronger effect on one of the bladder cancer cell lines than on normal uroepithelial cells. A favourable trend of Mumio was observed in the second tested cancer cell line with prolonged exposure indicating potential long term administration benefits. These promising results form the basis for further investigations of potential applications of Mumio in the treatment and prevention of bladder cancer (Fig. 1).

## Conclusion

Our results indicate that more interest should be given to the evaluation of Mumio’s potential use for cancer treatment. In can be potentially difficult to analyse the distribution of Mumio to various tissues because of the complex composition. Due to these considerations, we suggest to further investigate intravesical application together with long term oral administration of Mumio for bladder cancer treatment. Moreover, long term administration of Mumio was found to be save in an animal model and clinical studies did not show significant adverse events after Mumio administration^9, 18^. However, it is important to consider the depletion of insoluble mineral salts in order to limit the risk of bladder stones and irritation of bladder wall, which could create a niche for implantation of tumour derived cancer cells.

## Materials and methods

### Cell lines

Human normal uroepithelial (SV-HUC-1), human transitional bladder cancer (T24) and human grade II urinary carcinoma (5637) cell lines were purchased from American Type Culture Collection (ATCC, USA). T24 cells were cultured in DMEM/Ham`s F-12 medium (Corning, USA) with 10% foetal bovine serum (FBS) supplemented with 5 μg/ml amphotericin B, 100 μg/ml streptomycin and 100 U/ml penicillin. SV-HUC-1 cells were cultured in F-12 K medium (Corning, USA) with same supplements. 5637 cells were cultured in RPMI-1640 medium (Corning, USA) with same supplements. All cell lines were grown in plastic tissue culture T-flasks 75 cm^2^ (Corning, USA) at 37 °C and 5% CO_2_. Cell lines were authenticated by American Type Culture Collection.

### Preparation of the Mumio solution

Mumio solution was prepared from commercially available 200 mg pills of Mumio Altajské (OOO FARMGRUP, Russia). Pills were dissolved in 10 ml of medium and centrifuged (500×*g*, 10 min, RT) to precipitate insoluble inorganic components. The supernatant was filtered through a 0.22 µm syringe filter (Merck Millipore, USA) and the stock solution (20 mg/ml) was further diluted in culture medium to obtain selected concentrations (200, 500, 800, 1000 µg/ml).

### MTT assay

Cell viability was assessed via the MTT assay. Cells were seeded on flat bottom 24-well plates (Corning, USA) at a density of 10,000 cells/cm^2^. After 24 h, cells were treated with varying Mumio concentrations based on a pilot study (200, 500, 800, 1000 μg/ml) for 24, 48 and 72 h. Cells cultured in standard culture medium were used as a control. After treatment, 500 μl of MTT solution (1 mg/ml; Sigma-Aldrich, USA) was added to each well and incubated for 2 h at 37 °C in dark. Formazan crystals were dissolved in dimethyl sulfoxide (DMSO, POCH, Poland) and the absorbance was measured at 570 nm (characteristic wave length) and 655 nm (reference wave length) using a Varioskan LUX Plate Reader (ThermoFisher Scientific, USA). The reduced viability was determined through comparison of absorbance in drug-treated wells to the control. These results were used to calculate the LC that caused death of 10, 50 and 90% cells—LC_10_, LC_50_ and LC_90_. These values are considered to represent the main cytotoxic characteristics of a drug. The LC_10_ is considered the first dose causing cytotoxicity, LC_50_ is a measure of cytotoxicity and LC_90_ is the lethal dose. In further experiments these concentrations were used to analyse the mechanism of action of Mumio.

### Selectivity index

The selectivity index (SI) is a measure for differential activity of a drug against cancer cells in comparison to normal cells. To asses SI value the following formula was been used:

$$Selectivity\,index\left( {SI} \right) = \frac{{LC_{50} \,in\,normal\,cell\,line}}{{LC_{50} \,in\,cancer\,cell\,line}}$$

Values greater than 2.0 indicate selective cytotoxicity according to Koch et al*.*^26^*.*

### Real-time cell growth analysis

Real-time cell growth analysis was performed with xCELLigence RTCA DP (ACEA Bioscience, USA) in order to confirm the calculated LC_10_, LC_50_ and LC_90_ values obtained in the MTT assay. Cells were seeded on an E-Plate 16 (ACEA Bioscience, USA) and incubated in normal cell culture medium for 24 h. Mumio was added at concentrations corresponding to its LC values. Cell growth and adhesion were monitored every 30 min for 24, 48, and 72 h. Electrical impedance was measured to monitor cellular changes and is presented as a cell index (CI). Cell index values can be used to monitor cell viability, number, morphology, and adhesion in a number of cell-based assays. Medium without cells served as the background control, and cells cultured in normal medium were used as the control.

### F-actin staining

Cells were seeded on 18 mm glass coverslips (Thermo Scientific, USA) placed in 12-well plate (Falcon, USA). After 24 h, cells were exposed to Mumio at their 24 h LCs. After drug exposure, the cells were washed twice with PBS (Corning, USA), and fixed in 2% methanol-free paraformaldehyde (Polysciences, USA) for 15 min. Cells were then permeabilised with 0.1% Triton-X 100 in PBS for 15 min and then washed twice with PBS. Followed by staining (30 min, RT) with Phalloidin-Alexa Fluor 488 (Thermofisher Scientific, USA). Counterstaining was performed with DAPI (15 min, RT) (Sigma-Aldrich, USA). Stained coverslips were mounted with Aqua-Poly/Mount (Polysciences, USA) on glass slides and investigated with an Olympus SpinSR10 confocal microscope (Olympus, Japan).

### Cell cycle analysis

Cell cycle analysis with Tali® Cell Cycle Kit (Thermo Fisher Scientific, USA) was performed after cells were exposed to Mumio at their 24 h LC_10_, LC_50_ and LC_90_ concentrations. Detached cells were fixed in 70% ethanol at − 20 °C, and kept in these conditions for at least 24 h prior to staining. Cells were washed to remove ethanol and then stained with PI/RNAse solution for 30 min at room temperature. Stained cells were analysed by flow cytometry with BD FACSCanto II (BD Biosciences, USA). At least 10 000 scatter gated events were recorded from each sample. Data was analysed with FlowJo v10.3 Software (Becton Dickinson, USA; https://www.flowjo.com/solutions/flowjo). Cell number analysis of G0/G1, S and G2/M phase cells was performed using the Watson (pragmatic) model.

### Apoptosis detection

Apoptosis was assessed via the quantification of phosphatidylserine (PS) on the outer cell membrane. After 24 h of drug exposure to LC concentrations, cells were detached and stained with the FITC Annexin V Apoptosis Detection Kit II (BD Biosciences, USA). Staining was performed according to the manufacturer instructions. Cells stained with Annexin V-FITC and propidium iodide were analysed by flow cytometry using the BD FACSCanto II (BD Biosciences, USA). At least 10 000 scatter gated events were recorded for each sample. Obtained data were analysed with BD FACSDiva Software v. 8.0.1 (BD Biosciences, USA; https://www.bdbiosciences.com/en-pl/products/software/instrument-software/bd-facsdiva-software).

### Statistical analysis

Each experiment was performed at least in triplicate. Mean cell viability was expressed as a percentage relative to the control. All data are presented as means ± standard deviation (SD). Statistical analysis was performed using one-way ANOVA with Tukey post-hoc (for cell viability) or two-way ANOVA with Sidak’s post-hoc (for grouped analysis) using GraphPad Prism 8 (GraphPad Software, USA; https://www.graphpad.com).

## References

[1] Carrasco-Gallardo C, Guzman L, Maccioni RB (2012). Shilajit: A natural phytocomplex with potential procognitive activity. Int. J. Alzheimer's Dis..

[2] Cheryll W (2013). Medicinal Plants in Australia.

[3] Garedew A, Feist M, Schmolz E, Lamprecht I (2004). Thermal analysis of mumiyo, the legendary folk remedy from the Himalaya region. Thermochim. Acta.

[4] Ghosal S (1991). The need for formulation of shilajit by its isolated active constituents. Phytother. Res..

[5] Aiello A (2011). Mumijo traditional medicine: Fossil deposits from Antarctica (Chemical composition and beneficial bioactivity). Evid. Based Complement. Altern. Med..

[6] Cornejo A, Jimenez JM, Caballero L, Melo F, Maccioni RB (2011). Fulvic Acid inhibits aggregation and promotes disassembly of tau fibrils associated with Alzheimer's disease. J. Alzheimers Dis..

[7] Ghosal S (1988). Anti-ulcerogenic activity of fulvic acids and 4′-methoxy-6-carbomethoxybiphenyl isolated from shilajit. Phytother. Res..

[8] Schepetkin I, Khlebnikov A, Kwon BS (2002). Medical drugs from humus matter: Focus on mumie. Drug Dev. Res..

[9] Velmurugan C, Vivek B, Wilson E, Bharathi T, Sundaram T (2012). Evaluation of safety profile of black shilajit after 91 days repeated administration in rats. Asian Pac. J. Trop. Biomed..

[10] Windmann W (2005). Mumijo, Das schwarze Gold des Himalaya.

[11] Agarwal SP, Khanna R, Karmarkar R, Anwer MK, Khar R (2007). K. Shilajit: A review. Phytother. Res..

[12] Galimov, E. M., Kodina, L. A., Vlasova, L. N., Velyukhanova, T. K. & Bazilevskaja, O. L. Geochemistry of mummiyo. *Geokhimiya* 1494–1505 (1986).

[13] Yance DR (2013). Adaptogens in Medical Herbalism: Elite Herbs and Natural Compounds for Mastering Stress, Aging, and Chronic Disease.

[14] Frolova L, Kiseleva T (1996). Chemical composition of mumie and methods for determination of its authenticity an quality. ChemPharm J.

[15] Schepetkin IA, Xie G, Jutilla MA, Quinn MT (2009). Complement-fixing activity of fulvic acid from Shilajit and other natural sources. Phytother. Res..

[16] Cancer, I. A. f. R. o. *GLOBOCAN. Cancer Incidence and Mortality Worldwide.* http://gco.iarc.fr/ (2018).

[17] Society, A. C. *Bladder Cancer—Causes, Risk Factors, and Prevention.* https://www.cancer.org/cancer/bladder-cancer/causes-risks-prevention.html (2019).

[18] Sadeghi SMH (2020). Efficacy of momiai in tibia fracture repair: a randomized double-blinded placebo-controlled clinical trial. J. Altern. Complement. Med..

[19] Andriukhova NN (1997). The treatment of benign prostatic hyperplasia using the Mumie-Vitas preparation. Likars'ka sprava.

[20] Sui XB, Lei LM, Chen LX, Xie T, Li X (2017). Inflammatory microenvironment in the initiation and progression of bladder cancer. Oncotarget.

[21] Khanduja KL, Gandhi RK, Pathania V, Syal N (1999). Prevention of N-nitrosodiethylamine-induced lung tumorigenesis by ellagic acid and quercetin in mice. Food Chem. Toxicol..

[22] Thresiamma KC, George J, Kuttan R (1998). Protective effect of curcumin, ellagic acid and bixin on radiation induced genotoxicity. J. Exp. Clin. Cancer Res..

[23] Pant K (2016). Mineral pitch induces apoptosis and inhibits proliferation via modulating reactive oxygen species in hepatic cancer cells. BMC Complement. Altern. Med..

[24] Thawatchai P, Juree C, Penpun W, Chutima L, Thaksin S (2008). Some biological activities and safety of mineral pitch. Silpakorn Univ. Sci. Technol. J..

[25] Jafari M (2019). Antioxidant, cytotoxic and hyperalgesia-suppressing activity of a native Shilajit obtained from Bahr Aseman mountains. Pak. J. Pharm. Sci..

[26] Koch A, Tamez P, Pezzuto J, Soejarto D (2005). Evaluation of plants used for antimalarial treatment by the Maasai of Kenya. J. Ethnopharmacol..

